# Cross-national Comparisons of Intergenerational Relations between Older Parents and Adult Children

**DOI:** 10.1093/geroni/igaf122.1750

**Published:** 2025-12-31

**Authors:** BoRin Kim, Ke Li

**Affiliations:** University of New Hampshire, University of New Hampshire, New Hampshire, United States; Ohio University, Athens, Ohio, United States

## Abstract

This study aims to identify and compare various patterns of intergenerational relations as well as to examine their associations with depressive symptoms among older adults across three countries, which have different family cultures and policies. Guided by the intergenerational solidarity framework, the present study explored the multidimensional portrayal of intergenerational relations including structural, associational, functional, and normative solidarity. Data came from the 2018 harmonized datasets of the Gateway to Global Aging from China, South Korea, and the United States. The sample was restricted to respondents over 65 years with at least one child, and one respondent per household was selected (China N = 4,368; South Korea N = 3,093; the US N = 2,153). Latent Profile Analysis was used to identify patterns of intergenerational relations with nine variables including the number of sons and daughters, distance to the geographically closest child, contact frequency (face-to-face and remote), and physical and financial help from/to children. OLS regressions were used to analyze the associations between intergenerational relations and depressive symptoms among older parents. Five clusters were identified as the most optimal classification: Disconnected, Distant-Interacted, Interdependent, Close-Helping, and Coresiding-Dependent Parents. The proportions and the characteristics of each intergenerational profile significantly differed across countries. The associations between clusters and depressive symptoms also varied. This study confirmed that intergenerational relations are influenced by the interplay between micro-level individual and family attributes as well as macro-structural policies and cultures. Our findings offer valuable insights into cultural norms, societal structures, and policy implications related to family dynamics and aging.

